# “When you first walk out the gates…where do [you] go?”: Barriers and opportunities to achieving continuity of health care at the time of release from a provincial jail in Ontario

**DOI:** 10.1371/journal.pone.0231211

**Published:** 2020-04-10

**Authors:** Catherine Hu, Jessica Jurgutis, Dan Edwards, Tim O’Shea, Lori Regenstreif, Claire Bodkin, Ellen Amster, Fiona G. Kouyoumdjian

**Affiliations:** 1 McMaster University, Hamilton, Ontario, Canada; 2 Lakehead University, Thunder Bay, Ontario, Canada; University of North Carolina at Chapel Hill, UNITED STATES

## Abstract

We aimed to explore continuity of health care and health barriers, facilitators, and opportunities for people at the time of release from a provincial correctional facility in Ontario, Canada. We conducted focus groups in community-based organizations in a city in Ontario, Canada: a men’s homeless shelter, a mental health service organization, and a social service agency with programs for people with substance use disorders. We included adults who spoke English well enough to participate in the discussion and who had been released from the provincial correctional facility in the previous year. We conducted three focus groups with 18 total participants. Participants had complex health needs on release, including ongoing physical and psychological impacts of time in custody. They identified lack of access to high quality health care; lack of housing, employment, social services, and social supports; and discrimination on the basis of incarceration history as barriers to health on release. Access to health care, housing, social services, and social supports all facilitated health on release. To address health needs on release, participants suggested providing health information in jail, improving discharge planning, and developing accessible clinics in the community. This pilot study identified opportunities to support health at the time of release from jail, including delivery of programs in jail, linkage with and development of programs in the community, and efforts to support structural changes to prevent and address discrimination. These data will inform ongoing work to support health and continuity of care on release from a provincial correctional facility.

## Introduction

Compared with the general population in Canada, people who experience incarceration have worse health, with high rates of mental illness, infectious disease, premature mortality, and poor social determinants of health [1]. This excess burden of disease influences population health through transmission of infectious disease, costs of healthcare and recidivism, impacts on family and community health, and impacts on public safety [2–5]. People who are Indigenous, who are Black, and who have low socioeconomic status are overrepresented in Canada’s prison population [1,5,6]. This overincarceration is produced by legacies and ongoing practices of colonization, racism, and systemic economic inequality, which create disproportionate health challenges among these communities [7–12]. Improving health in people who experience incarceration is therefore an important pathway to improving public health and health equity in Canada [2].

The period after release from custody is particularly challenging to health, as indicated by increased risks of morbidity and mortality [13–16]. People face urgent issues on release from jail and prison, including finding housing and employment, reconnecting with family, and meeting conditions of release [17–23], which may compete with addressing health and health care needs [24]. In the US, lack of health insurance poses a significant barrier to accessing health care after release, especially in states where Medicare and Medicaid are revoked upon arrest [18–20,23, 25–27]. People may face discrimination relating to their criminal record, including from health care providers [17,26,28]. Finally, inadequate discharge planning in custody may contribute to difficulty navigating community services on release [20,22]. In this context, stakeholders have identified continuity of health care and community re-integration as priorities for prison health research in Canada [29].

There is growing international research on transitional programs to link people with health care and other services in the community after release from prison and jail, which range from providing pamphlets and telephone calls to specialized clinics and intensive case management, and have shown success in engagement and retention in health care in the community [30–36]. Such promising programs may have value for people on release from provincial correctional facilities in Ontario, given provincial and local data showing that one third or more of people who experience imprisonment do not have access to primary care in the community [37,38].

As we consider strategies to support health and access to health care in people on release from a provincial correctional facility in southern Ontario, we need current, local data on the experiences and perspectives of people who have recently been incarcerated [39]. Data from the US and Australia may not be generalizable to the Canadian setting, given national differences in criminal justice systems, social and economic policy, and health care systems, and data are limited on health challenges and opportunities in the post-release period for people in Canada [24,36,40,41]. Further, programs and services may vary substantially by province, region and correctional facility, and needs and opportunities change over time based on service availability and population health issues, for example, the current opioid overdose crisis [42].

Our study objective was to explore continuity of health care and health barriers, facilitators, and opportunities for people at the time of release from a provincial correctional facility in Ontario, Canada.

## Methods

We used a qualitative design, in which we conducted focus groups with people recently released from a provincial correctional facility in Ontario, Canada. Provincial correctional facilities hold people who are admitted to custody without sentencing and people sentenced to less than two years in custody [43]. Recent data indicate that the median length of stay for people in provincial correctional facilities in Ontario is 10 days with an interquartile range of 3 days to 52 days [13]. For Ontario residents, hospitalizations and medically necessary physician services including primary care and emergency department visits are paid for through the public health insurance system, the Ontario Health Insurance Plan, including while in provincial correctional facilities. Health care in provincial correctional facilities is administered by the Ministry of the Solicitor General.

*A priori*, we planned to conduct between three and six focus groups with the goals of exploring diverse perspectives and achieving saturation. We recognized that we might not achieve saturation with this number of focus groups, but we set this as the target number in consideration of available funding. Our interest was in continuity of health care and continuity of health, defined by the World Health Organization as: “a state of complete physical, mental and social well-being and not merely the absence of disease or infirmity” [44]. We therefore aimed to include people who may face particular challenges to continuity of care in the post-release period, such as people with mental illness and people with substance use disorders. We conducted one focus group at each of three community-based organizations in September and October 2018: a men’s homeless shelter, a mental health service organization, and a social service agency providing voluntary and court-ordered programs for people with substance use disorders. The focus group guide (S1 File) was developed by three project team members (Catherine Hu, Jessica Jurgutis, and Fiona Kouyoumdjian) based on the study objectives and issues identified in previous research [36].

We included adults (aged 18 and older) who had been released from the provincial correctional facility within the past 12 months, who consented to participate, and who spoke English well enough to participate in the focus group, and we used convenience sampling. At each site, organizational staff posted information about the study and shared information with potentially eligible clients about the study and the scheduled time for the focus group. Focus group facilitators assessed capacity to consent at the time of reviewing the information and consent forms by determining whether potential participants understood the nature of the research, including the goals of the research, risks and benefits. All participants signed up for the focus groups through case workers, program facilitators, and shelter staff at the respective organizations, and no potential participants were deemed to be lacking or at risk for lacking capacity to consent. Though some participants were accessing services for support with mental illness and substance use, it is also important that this research did not inappropriately exclude participants on the basis of additional forms of social marginalization such as health status as long as they were capable to consent.

At the scheduled time, two focus group facilitators met with interested participants. The focus groups were co-facilitated by two women: Jessica Jurgutis, a PhD candidate with extensive experience in qualitative research, and Catherine Hu, an undergraduate student who was coached by Jessica Jurgutis and self-trained in focus group facilitation for the purposes of the project. Neither facilitator had a prior relationship with the study participants. The facilitators did not provide information on their personal goals or reasons for conducting the research, or on their ideas or assumptions regarding the topics discussed. The facilitators completed the informed consent process, obtaining written consent for participation and for audio recording of the focus group. They led a semi-structured discussion based on a guide, which included questions on challenges at the time of release from custody, barriers to addressing health issues on release, barriers to accessing primary care on release, gaps in services in the jail and in the community, and opportunities to support the transition to the community before, during, and after release (S1 File). The focus groups lasted between one and one and a half hours. No one was present for the focus groups besides the participants and researchers. The focus groups were audio recorded and the facilitators also made field notes. Each participant was provided $30 and two bus tickets as compensation for participation.

We transcribed data verbatim from the focus groups. We used NVivo software to conduct an inductive qualitative content analysis [45], meaning that we reviewed data to identify emerging themes rather than using data to test a specific theory. Three researchers (Jessica Jurgutis, Catherine Hu, and Fiona Kouyoumdjian) participated in coding. Two people reviewed each transcript for themes, and we developed and revised the coding framework iteratively as we coded the data. Our coding tree included seven themes: (1) experiences in custody, with subthemes challenging environment in jail, health care in jail, and differences between jail and the community, (2) thoughts and feelings regarding experiences in custody, (3) challenges on release, with subthemes housing, ongoing criminal justice system involvement, employment, broken social bonds, health care needs, cycle of crime, pleas deals and impact, and taking care of basic needs, (4) thoughts and feelings regarding challenges on release, (5) barriers and facilitators of success on release, with subthemes barriers to success, facilitators of success, and opportunities to support success, (6) thoughts and feelings regarding barriers and facilitators of success on release, and (7) value and importance of health, with subthemes in jail and after release. We used the thematic analysis to form a set of conclusions about our research question.

We did not return transcripts to participants for comment or correction, and participants did not provide feedback on the findings.

The study protocol was approved by the Hamilton Integrated Research Ethics Board.

## Results

We conducted three focus groups with a total of 18 participants. Participants were adults between the ages of 20 and 69; we did not collect data on race, gender, length of time spent in custody, or whether participants were on probation or parole in the community.

In this report, we focus on focus group content about barriers, facilitators, and opportunities for health. We placed additional emphasis during analysis on the feelings that participants expressed as a means to better understand issues that resonated among participants, and to acknowledge participants’ emotional experiences.

### Barriers to health after release

#### Health issues and health care needs

Participants had diverse health problems upon release from jail, most of which related to mental health, substance use, or pain management. Substance use and recovery were concerns for many participants. As one person shared: “A lot of us get stuck [while working to rebuild a life] …and we can’t go forward. Sometimes, we just […] start drinking again, and we start doing drugs again.” In turn, relapse could lead to getting into “the same old cycle [of crime involvement], over again” and going “back to the old ways [to] support your habit.” Many participants had chronic pain, which they managed with prescribed medication.

After release, participants experienced physical and psychological impacts of their time in custody. Some had decreased physical fitness due to the lack of space and exercise opportunities in the provincial correctional facility. Some had lower energy, and they described “getting tired easy” and “[gaining] weight,” which prevented them from being as active as they wanted after release. One person stated that changes in jail to their medications made them feel “pain,” “fear,” and “[thoughts of] suicide,” which persisted “even when [they got] out.” Others described trouble relating and connecting to others in the community due to the social isolation they experienced in jail, and feeling “institutionalized” by their time in jail—in other words, they continued thinking the way they used to in jail even after release, including how they “[dealt] with people in public, cleanliness…everything.”

Participants reported varied experiences with accessing health care in the community. Some did not have a Family Physician and had trouble accessing a Family Physician. Another participant had been unable “to get mental health help for the past few months” since they didn’t have a Family Physician, which they needed for a referral: “You got to go through a doctor, right? How many people have a family doctor? Especially when you get out. You don’t have a family doctor when you get out again.” Another participant described their experience finding mental health care after jail as:

…not having the simplest clue on where to go, because it’s not like you have big signs, like you know what I mean? Come here if you want this, like, you know? So for—to actually find some of these places, it’s pretty difficult for me…

Another issue was quality of care on release. A participant described the quality of the health care they and others could access as “the lowest of the low.” Many people reported discrimination from health care providers based on their history of incarceration. One participant said, “[health care providers] automatically judge me, because I was in jail.” Another said, “they automatically assume that you’re a junkie, or you know, you’re only trying to scam them to get what you want, right?” Another person said that their doctor took them off their pain medication upon finding out about their charges.

Participants described substantial barriers on release to accessing medications they were taking prior to admission. One person stated that “[people after release from jail] have the hardest time even going back to their regular doctors and getting it back to the same script.” Not having access to medications impacted other aspects of life, as noted by one person who needed “painkillers… [so they could] just have a normal routine, and go back to work.” For some this reflected changes to medications in jail; several participants had medications stopped, switched, or reduced in jail, with one claiming: “If you’re on six painkillers a day on the outside, they’ll give you two.” This led to community-based health care providers refusing to prescribe prior treatments when “they find out that you weren’t on your meds anymore.” People would then need to “run around” or go through a “rigamarole” before restarting medications, such as having to see a specialist or undergo repeat testing.

#### Housing, employment, and social services

Lack of access to housing, employment and social services affected participants’ health on release, as one participant described:

You’re out, and you got nothing to do, you can’t find a place to live, you’re stuck […] and that’s where you fall behind.

Several participants reported losing “everything” upon entering jail and needing to “start again.” One person explained:

It’s an up—it’s a very big hill [after release]. Especially when you lose all your stuff, and your place, and your… not even have clothes that were yours, when you got out.

Many described their experiences locating affordable housing as one of the most significant obstacles they faced on release, due to discrimination and a lack of access to information, resources, and support capacity within community agencies. Finding a new home was challenging for people whether they were doing it independently or with support. One participant described their experience at a shelter as follows:

…they don’t have a working computer. All they did was get a newspaper in once a day. It was totally up to you to find a place. […] And anything they did post was all out of everybody’s rent. Like, what do you think? It was ridiculous.

Participants experienced similar struggles with employment. They described being turned away from opportunities or “brush[ed] right off” due to their criminal record. Ongoing involvement with the criminal justice system also created stress, with participants worried about upcoming court dates and meeting conditions of release.

It was also challenging to access social services in the community. Participants expressed frustration about the time required and the “hoops” they had to jump through to access services such as welfare or disability insurance. One person said it was necessary to “put your nose to the grindstone to get help,” and to fix the “loose ends” such as debt and eviction caused by their time in custody.

Several people described how discharge planning in custody did not prepare them well for release, as it was brief and limited, and did not focus on health needs. Some plans made in custody fell through as soon as people returned to the community, as one person explained:

The social worker or whatever will meet with you a couple times here and there. Like, kind of work out a little plan? But sometimes that plan just…the plan just doesn’t work all the time. […] Yeah, you have a goal, you have a plan. But once you walk out those gates, that plan just—poof.

### Facilitators of health after release

#### Access to services and supports

Participants described how health care professionals supported their health through prescribing medications, helping them get “linked up” with services, and ongoing positive relationships:

I find what helps me is I get my methadone every day. And even something as simple as that gets me out of the house, and—so I can even talk to a doctor, or even briefly for like, a few minutes. It’s not like you go into depth or detail with the methadone doctors, but just to be able to actually check in with a doctor every few days is just…it’s nice. And I think methadone has really helped me with that. Um, it’s kind of getting me out of my shell.

They also described how social services staff helped people find housing and connect with other supports, and provided valuable health teaching, for example on how to manage medications and build motivation. A participant described how a program helped them make positive life changes such as finding a family doctor and attending a gym, while another commented on how the program staff helped to support them to continue in the right direction.

Many participants expressed the importance of support from family, friends, partners, and others in their social network. As examples, family and friends provided a place to stay, referred them to services, and offered advice and guidance. Participants shared how helpful it was to have someone who cared for them, talked to them, and supported them during what was often a difficult time. Those without many friends or family in the community spoke about how volunteering or attending peer support groups provided them with valuable social connections and helped them feel less alone.

### Emotional and psychological experience post-release

#### Negative impacts of incarceration

Participants described profoundly negative psychological impacts of incarceration on release. They described feeling “lost” after release from the jail from having “everything” taken away. As one participant explained:

You walk out, when you first walk out the gates, it’s like fuck, where do I go? What do I do? Who do I call? Especially when you’re wearing the blues, you know?

Those who had been incarcerated multiple times explained that everything they managed to rebuild got “torn down again” each time they returned to jail. In health care, housing, employment, and day-to-day life, participants described stigma and shame relating to their criminal record. They described a sense of loss at how demoralizing it was to rebuild their lives and start over again after release, and feeling like they were “stuck” or falling behind.

Participants described a sense of having to “take care” of themselves and be self-sufficient in the post-release period, with one describing a sense of having to “be your own doctor.” These challenges took a toll on some participants, which one person described as “getting depressed, and overwhelmed.”

#### Positive experiences on release

Participants also reported positive experiences with having people who helped them reintegrate into the community, and access to the care they needed. Many people said they felt lucky to have supports and access to services. They described not knowing where they would be or what they would have done without the help they had, or thinking that their situation would have been much worse than it had been. Some participants felt supported and cared for, and not alone. One person commented that they were finally “getting the proper help that [they] need,” which they were unable to access in jail.

Participants indicated that high quality care and relationships with community health care providers played an important role in helping them access meaningful care and support. For instance, some expressed appreciation for doctors who were “really taking good care of [them]” and with whom they were able to “check in” on a regular basis. Having an established relationship with a health care provider facilitated continuity of prescribed medication after release. As one person described: “[I]f you got out of jail, and you were on methadone, and you went back to the methadone clinic you were on, you’d be on it right away.”

Health care providers also helped people get “linked up” with social services. Participants emphasized how helpful it was for health care providers to be non-judgemental, attentive, respectful, and accessible, for example co-located with other health and social services. One person noted the value of a supervised consumption site, saying “how much…good [the site] does for people” and that harm reduction sites such as this are “a lifesaver” and went on to say:

… the doctors there, they’re so nice. And they make me feel so much better about myself whenever I go there. I mean, I can sit and chat and just have a conversation about details, or how my day’s going, or about things I’m worried about, or what things I could do better.

They emphasized the importance of having people in their lives who made them feel valued and who motivated them to take care of themselves, which they would not have done on their own. They felt that the services they accessed gave them knowledge and guidance that “rehabilitated” them and helped keep them on track in their re-integration process.

### Opportunities to support health after release

#### Discharge planning and health teaching in custody

Participants identified that more services were needed “inside and outside,” and that providing services only in the community after release “might be too late.” Across groups, they commented that it would be valuable to improve discharge planning in custody, including by having a more explicit focus on health:

Teach us—show people where to go, what to do, you know, in their last couple days in. Then […] people can make their choices as they leave, right?

Like, with all the barriers that there might be when we get out. Because I did my discharge planning with [a community provider], but she didn’t really…touch on anything like, you know? It was just, do you have housing? And then if you didn’t, um, they wouldn’t help you out with housing, but they’d help you get set up with welfare, and that was it, I think.

Several people identified the importance of providing information in custody prior to release, including health education and information on community resources. This included suggestions to provide literature on community-based programs and resources, facilitated information sessions, and specific information about relevant services such as addictions programs and counselling. Many emphasized that in the current conditions, anything would be better than the status quo. As one participant clarified, “what they teach us in here [at a community-based treatment program for people with substance use disorders], they should teach it in jail,” especially since that teaching had assisted with rehabilitation.

#### Resources in the community

Many participants indicated that community services and support needed to be more robust and adaptive to assist people in responding to the instability and changing circumstances that they face upon release, for example, when a plan “doesn’t work” or when goals need to be re-evaluated because of the everyday challenges faced. Across focus groups, several people agreed it would be valuable to have a clinic where recently released people could access care:

…there is no […] special clinic for released inmates, right? There isn’t any. It’s like, people that come out of jail automatically get sent over there, get a total physical, make sure they’re hundred percent, and whatever is wrong with them, to start putting them on a treatment. I haven’t seen one of those.

Participants emphasized that clinics and other service providers should “make adjustments” to provide appropriate and accessible care so that people “would use it” on release. They suggested having clinics located near correctional facilities. In this kind of facility, “you could get more help, when you get out […] because it would lead you in the direction of mental health, medical, […] housing, peer support groups, maybe, too.” Given the substantial burdens faced by those leaving custody, forms of support such as this could help prevent negative health impacts of trying to “do too much at once” because “everything will go wrong.” Other participants identified that such a clinic could “teach people” by having an educational component, providing information, and helping people develop a “treatment plan.”

## Discussion

In this qualitative study, participants described diverse and complex health needs on release from a provincial correctional facility. They had challenges finding good medical care, housing, and employment, and accessing ongoing treatment. They encountered discrimination related to their criminal record in accessing health care services, housing, and employment. Participants described how incarceration and the post-release period substantially impacted their health, and many felt lost, stigmatized, demoralized, and overwhelmed on release. Access to health care, social services, and social relationships supported participants’ health, and made them feel lucky and cared for. They identified the need for better discharge planning and for information on health issues and community resources while in custody, and access to a clinic at the time of release.

Our finding that mental health, substance use, and pain management were major concerns for people on release from custody is consistent with other research [20,21,25,28,46]. Similarly, other studies have also identified stigma and discrimination [17,19–21,26,28], competing priorities such as housing and employment [17–23], lacking access to health care and other services [17,21,22,25,26], and lack of continuity of care on release [17,21,22,24,25,40,47] as barriers to health on release. Several studies corroborate our findings regarding facilitators of health on release, including support from family and friends, access to social services, convenient and accessible health services including for opioid agonist treatment such as methadone, and service providers [17,19,23,26,40]. Other studies also identified facilitators not discussed in the current study, such as support from parole officers and motivation to care for their children among people who were parents [19,23].

This study has several potential limitations. We did not achieve saturation. We concluded the study after conducting three focus groups, as we had spent the funds that were available for this pilot study, and if we had conducted more groups we would not have been able to compensate additional participants or pay research staff. While our study findings may not be generalizable to all people who experience incarceration in provincial correctional facilities, we expect that the results would be relevant for a large proportion of people who experience incarceration, especially given the high prevalence of mental illness, substance use disorders, and precarious housing and homelessness in this population [1,48,49]. We did not collect data on the length of time people spent in custody or on whether participants were supervised through the correctional system after release, and these factors may be relevant to experiences of health and health care on release. There was limited ability to apply an intersectional lens in our analysis due to the small sample size and lack of formal data collection on race, gender, or other factors of potential interest. Given that women, people who are gender non-conforming, people of colour, and Indigenous persons have unique health experiences shaped by structural determinants of health during and after incarceration and more generally, being able to capture these experiences may be an area of priority in future research on health experiences in the transition between the jail and the community [18,50,51].

Study strengths are that we included people who may be more likely to have health needs after release from custody, namely people with mental illness, people with substance use disorders, and people who were homeless or precariously housed. This study provides contemporary data from the Canadian context, as one of a limited number of qualitative studies conducted in Canada on health needs on release [24,40,41], and one of only two such studies conducted since 2010 [24]. The findings demonstrate that even in the context of universal health insurance, recent primary care reform to enhance access and quality [52], bolstered treatment and harm reduction programs for people who use illicit drugs in the context of the opioid overdose crisis [42], and the implementation of innovative court models such as drug and mental health courts, people who experience imprisonment continue to face major challenges to health and continuity of health care at the time of release.

Dealing with physical and mental impacts of time in custody and urgent needs such as finding housing and employment, people post-release may need to work harder than the average person in the general population to try to meet their health care needs, and given issues of health care access and quality, they may be less likely to be able to meet their needs. Recognizing this unique set of barriers to health at the time of release may lead to reframing how we consider obligations for the State and for health care and social services to attend to the needs of this population at this vulnerable time; a greater focus on health at this transition may redress the harms of incarceration and support people as they work to improve their health, which could improve both health and health equity. Population-level opportunities include enhancing integration of prison and community health care services, consistent with the principles articulated in the World Health Organization’s Moscow Declaration [53], and explicitly including a focus on people who experience imprisonment in the development of social and health services [54].

In addition, we suggest a focus on individual-level strategies to improve health on release, which should be developed in collaboration with people with lived experience and with evaluation of acceptability, accessibility, and effectiveness. Building on evidence from transitional programs [30–36], concrete solutions may include comprehensive and patient-focused discharge planning before release from jail, the provision of information in jail and in the community on health issues and community-based services, and enhancement of programs to link people in custody with services in the community, including primary care. After release, emphasis should be placed on supports that have the capacity to adapt and respond to people’s changing circumstances and needs, including harm reduction and substance use disorder treatment programs such as opioid agonist treatment programs. Other broader strategies to support health after release may include initiatives to protect people from discrimination in health care, housing, and employment due to criminal record, linking people with peer support networks and other sources of social support after release, and continuing efforts to improve access to and quality of health care within the jail. Supporting people building networks of health care, services, and supports may be key to assisting people’s health, continuity of care, and reintegration into the community.

## Supporting information

S1 Checklist COREQ (consolidated criteria for reporting qualitative research) checklist. (PDF)

S1 File Focus group guide. (DOCX)
