# Supplementary material for: “When you first walk out the gates…where do [you] go?”: Barriers and opportunities to achieving continuity of health care at the time of release from a provincial jail in Ontario
Source: PLoS One. 2020 Apr 10;15(4):e0231211. doi: 10.1371/journal.pone.0231211 (PMC7147766; doi:10.1371/journal.pone.0231211)
Supplement: S1 File — (DOCX) [file pone.0231211.s002.docx]

**S1 File. Focus group guide**

| Challenges at the time of release | 1. Do you feel that the time after release from custody has been challenging for you? Why or why not?  2. Do you believe these challenges affect your ability to take care of issues related to your health? (i.e. seeing a doctor, attending a specialist appointment, etc.)  3. Is it important for you to be able to take care of your health when you are released from jail?  4. Do you think those who have spent time in jail or prison face specific challenges related to the time they spent there?  5. What are some things that get in the way of making the choices you want to make about your health?  6. Do you currently have a primary care provider? Why or why not? |
| --- | --- |
| Thoughts on and experiences of challenges at the time of release | 1. What has your experience been accessing health care after release from custody?  2. Have you experienced or heard of others experiencing any difficulties in addressing health issues and/or accessing the services they need? If so, what kinds of challenges have you or they encountered?  3.a. [If you feel comfortable sharing with the group,] Were there any health issues that were brought to your attention or that began to be addressed in custody that you have not been able to follow up on since you’ve left jail?  3.b. If yes, what have been some things that have gotten in the way of following up and/or getting the care you need?  3c. Has anyone supported you in managing your health when you were released from jail? Who was that and how did they help?  4. Have you ever felt pressured to look after a health concern that was not a concern and/or priority for you? Please explain.  5. Have you ever felt like there was a health issue you wanted to take care of, but that it was too much time/energy/work to do so? Please explain.  6.a. Was having access to health care in jail important to you? Why or why not?  6.b. Was there ever a time that you did not get access to the health care you needed while in custody?  7.a. Is having access to health care now in the community important to you? Why or why not?  7.b. Can you think of a time recently where you were not able to access health care when you needed it? Please explain. |
| Strategies to improve access to primary care and transition to community after release | 1. What improvements could be made to enable better access to health care: In jail? In the community?  2. If you could change anything about your experience accessing health care in jail or in the community what would it be?  3. What do you think would help assist people in addressing their health care needs after release from custody?  4.a. Thinking about your experience currently, is there anything that would make your ability to access health care easier? Why or why not?  4.b. If you feel satisfied with how your health care needs are being taken care of currently, what about your current health care arrangement is working for you?  4.c. Have you had any bad or uncomfortable experiences accessing health care in jail or in the community?  5.a. Are there any health care issues facing people after release from custody that you think are especially important to find solutions for?  5.b. If so, what are the issues and what solutions would you propose?  6.a. Do you believe there could be better support for people being released from custody to assist with transition to the community?  6.b. If so, what do you think could be done to assist people in addressing their health care needs during this transition? |
| Wrap up | Is there anything else you would like to add about accessing health care in jail or in the community? |
